# Residual Risk Factors to Predict Major Adverse Cardiovascular Events in Atherosclerotic Cardiovascular Disease Patients with and without Diabetes Mellitus

**DOI:** 10.1038/s41598-017-08741-0

**Published:** 2017-08-23

**Authors:** Fang-Ju Lin, Wei-Kung Tseng, Wei-Hsian Yin, Hung-I Yeh, Jaw-Wen Chen, Chau-Chung Wu

**Affiliations:** 1 Graduate Institute of Clinical Pharmacy, College of Medicine, National Taiwan University, Taipei, Taiwan; 2 School of Pharmacy, College of Medicine, National Taiwan University, Taipei, Taiwan; 3 Department of Pharmacy, National Taiwan University Hospital, Taipei, Taiwan; 4 Department of Medical Imaging and Radiological Sciences, I-Shou University, Kaohsiung, Taiwan; 5 Division of Cardiology, Department of Internal Medicine, E-Da Hospital, Kaohsiung, Taiwan; 6 Division of Cardiology, Heart Center, Cheng-Hsin General Hospital, Taipei, Taiwan; 7 School of Medicine, National Yang-Ming University, Taipei, Taiwan; 8 Mackay Memorial Hospital, Mackay Medical College, New Taipei City, Taiwan; 9 Department of Medical Research and Education, Taipei Veterans General Hospital, Taipei, Taiwan; 10 Institute of Pharmacology, National Yang-Ming University, Taipei, Taiwan; 11 Department of Internal Medicine (Cardiology Section), National Taiwan University Hospital, Taipei, Taiwan; 12 Graduate Institute of Medical Education & Bioethics, College of Medicine, National Taiwan University, Taipei, Taiwan

## Abstract

A prospective observational study was conducted to investigate the residual risk factors to predict recurrence of major adverse cardiovascular events (MACE) in atherosclerotic cardiovascular disease (ASCVD) patients with a high prevalence under lipid-lowering therapy, particularly in the subpopulations of diabetic and nondiabetic individuals. A total of 5,483 adults (with a mean age of 66.4 and 73.3% male) with established coronary heart disease, cerebrovascular disease, or peripheral artery disease were identified from the T-SPARCLE multi-center registry. Of them, 38.6% had diabetes. The residual risk factors for MACE are divergent in these atherosclerotic patients with and without diabetes. In diabetic subpopulation, the risk of MACE was significantly increased with heart failure (HF), chronic kidney disease (CKD) stage 4–5 (vs. stage 1–2), without beta blocker use, and higher non-HDL-C, after controlling for covariates including statin use and the intensity of therapy. Increased LDL-C and TG levels were also associated with increased risk, but to a much less extent. Among nondiabetic individuals, HF, CKD stage 4–5, and history of myocardial infarction were the significant independent predictors of MACE. It is suggested that ASCVD patients with concomitant diabetes need stricter control of lipid, particularly non-HDL-C levels, to reduce cardiovascular risk when on statin therapy.

## Introduction

Individuals with atherosclerotic cardiovascular disease (ASCVD) are known to be at high risk for morbidity and mortality, and intensified lipid management and lifestyle modifications are essential and integral parts of secondary prevention^1, 2^. However, a considerable residual risk of cardiovascular disease (CVD) may still exist despite medical treatments^3^. While current guidelines emphasize the importance of lipid management, very few studies have inspected the risk of recurrent events among different ASCVD subpopulations and provide further guidance on how to lower the residual risk after lipid control. Diabetes mellitus (DM) is a particularly common comorbidity in ASCVD patients^4^, and the presence of DM increases the risk of mortality and cardiovascular complications in this population^5, 6^. The adverse influence of diabetes extends to all components of the cardiovascular system and different forms of CVD, such as microvascular and macrovascular complications affecting several vital organs including heart, brain, and kidneys. Risk factors related to cardiovascular complications may have different effects on the various manifestations of atherosclerosis. Studies have shown that patients with diabetes present widely varied susceptibility to coronary heart disease (CHD), in part depending on coexisting risk factors of atherosclerosis^7^. It is yet unclear after lipid control what critical risk factors lead to recurrent major adverse cardiovascular events (MACE) in ASCVD patients with DM, and whether these factors play an equally important role in ASCVD patients without DM.

Focusing on individuals with established CVD and treated for secondary prevention, the present study aimed to examine the clinical conditions and patterns of lipid management in ASCVD patients with and without DM in Taiwan. More importantly, we sought to investigate the residual risk factors for recurrence of MACE in these two subpopulations after lipid control, with the ultimate goal to improve clinical care and outcomes in these patients.

## Results

### Demographic and Clinical Features of Patients

A total of 5,483 ASCVD patients were included, with a mean age of 66.4 (SD 11.5), 73.3% male, and 89.2% with CHD (a flow diagram of patient enrollment is provided as online Supplementary Information). Among them, 38.6% (n = 2,117) had DM, mostly (>95%) type 2 diabetes. The baseline clinical characteristics of the DM and non-DM populations are shown in Table 1. Compared with those without DM (n = 3,366; 61.4%), diabetic ASCVD patients were older, more often female, with higher systolic blood pressure (SBP), and had a higher rate of coronary artery disease and prior intervention for coronary or peripheral artery disease (all *p* < 0.05). In addition, among patients with diabetes, important comorbidities such as hypertension, heart failure (NYHA class I-II), and chronic kidney disease were more prevalent, and the rate of using antiplatelet therapy, ARBs, ACE inhibitors and beta blockers were higher. At study enrollment, 69.5% of the diabetic ASCVD patients (vs. 65.5% in nondiabetic, *p* < 0.01) were treated with statins for secondary prevention against atherosclerotic events, with or without other lipid-lowering agents. The intensity of statin therapy was similar in treated patients irrespective of diabetic status. Only 3.5% diabetic and 3.2% nondiabetic patients were prescribed a high-intensity statin for secondary prevention of CVD with regard to the 2013 ACC/AHA guideline recommendations^2^. Fibrates were also more commonly prescribed in ASCVD patients with diabetes (6.9% vs. 4.8%, *p* < 0.01), considering their beneficial effects in reducing TG and increasing HDL-C levels. There were 63.5% and 4.6% of diabetic patients treated with oral hypoglycemic agents (OHAs) and insulin, respectively, and the mean HbA1c was 7.6%.

**Table 1:** Characteristics of the enrolled diabetic and nondiabetic patients.

Patient characteristics, n (%)	DM patients	Without DM patients	*p-value*
	n = 2,117	n = 3,366	
Age, mean ± SD	66.8 ± 10.8	66.0 ± 12.0	<0.05
Male	1478 (69.8)	2544 (75.6)	<0.001
BMI (kg/m^2^)			
<23	305 (15.2)	625 (19.5)	<0.001
23 −<27.5	968 (48.4)	1555 (48.6)	
27.5 −<32.5	590 (29.5)	859 (26.8)	
≥32.5	138 (6.9)	162 (5.1)	
Waist-hip ratio, mean ± SD	0.94 ± 0.08	0.93 ± 0.08	<0.001
Systolic BP (mmHg), mean ± SD	134.6 ± 18.6	131.7 ± 17.2	<0.001
Diastolic BP (mmHg), mean ± SD	75.9 ± 11.4	76.4 ± 11.2	0.13
Cigarette smoking status			
Past or current smoker	922 (43.6)	1482 (44.0)	0.78
No smoking	1191 (56.4)	1883 (56.0)	
History of atherosclerotic cardiovascular diseases			
Coronary artery disease	1915 (90.5)	2978 (88.5)	<0.05
Acute coronary syndrome	1676 (79.2)	2649 (78.7)	0.68
Myocardial infarction	1639 (77.4)	2623 (77.9)	0.66
Unstable angina	37 (1.7)	26 (0.8)	<0.01
Stable angina	239 (11.3)	329 (9.8)	<0.05
Cerebrovascular events	369 (17.4)	539 (16.0)	0.18
Ischemic stroke	301 (14.2)	425 (12.6)	0.09
Transient ischemic attack (TIA)	68 (3.2)	114 (3.4)	0.76
Peripheral arterial disease	44 (2.1)	71 (2.1)	1.0
Previous coronary or LEAD intervention	1170 (55.3)	1470 (43.7)	<0.001
Comorbidities			
Hypertension	1677 (79.2)	2359 (70.1)	<0.001
Heart failure (NYHA class I-II)	267 (12.6)	295 (8.8)	<0.001
Chronic kidney disease (GFR in mL/min)			
Stage 1–2 (greater than 60)	1259 (65.6)	2224 (75.5)	<0.001
Stage 3 (31–60)	563 (29.3)	674 (22.9)	
Stage 4–5 (30 or less)	97 (5.1)	48 (1.6)	
HbA1c (%), mean ± SD	7.6 ± 1.6	—	—
Lipid-lowering therapy	1574 (74.3)	2326 (69.1)	<0.001
With statin	1472 (69.5)	2203 (65.5)	<0.01
High-intensity	74 (3.5)	107 (3.2)	0.96
Medium-intensity	1178 (55.6)	1762 (52.3)	
Low-intensity	220 (10.4)	334 (9.9)	
With fibrate	146 (6.9)	162 (4.8)	<0.01
With antiplatelet therapy	1857 (87.7)	2854 (84.8)	<0.01
With ARBs or ACE inhibitors	1353 (63.9)	1897 (56.4)	<0.001
With beta blockers	1193 (56.3)	1688 (50.1)	<0.001
With OHAs	1344 (63.5)	—	—
With insulin	97 (4.6)	—	—

ACE = angiotensin-converting enzyme; ARB = angiotensin II receptor blocker; BP = blood pressure; GFR = glomerular filtration rate; LEAD = lower extremity arterial disease; NYHA = New York Heart Association; OHA = oral hypoglycemic agent.

### Lipid Profile and Goal Attainment

Diabetic ASCVD patients, as compared to those without diabetes, had lower LDL-C (mean ± SD: 92.7 ± 31.8 vs. 100.6 ± 35.1 mg/dL) and non-HDL-C (121.6 ± 37.5 vs. 126.9 ± 37.2 mg/dL), but higher TG level (152.2 ± 109.7 vs. 135.1 ± 83.4 mg/dL, all p < 0.001) than nondiabetic ASCVD patients (Table 2). Besides, 64.3%, 64.0%, and 62.1% of the diabetic patients attained a serum level of LDL-C < 100 mg/dL, non-HDL-C < 130 mg/dL, and TG < 150 mg/dL, respectively. When defining HDL-C goal as >40 mg/dL in men and >50 mg/dL in women, 46.7% of diabetic patients attained the goal. Among those without diabetes, the percentage of lipid goal attainment were only 55.5%, 58.9%, 70.0%, and 60.4% with regard to LDL-C, non-HDL-C, and TG, and HDL-C, respectively. In our cohort, merely 23.6% diabetic and 15.7% nondiabetic ASCVD patients achieved the LDL-C goal of <70 mg/dL based on the 2011 ESC/EAS guideline treatment target^1^.

**Table 2:** Lipid profile of the enrolled diabetic and nondiabetic ASCVD patients.

Lipid panel parameter (mg/dL), %*	DM patients	Non-DM patients	*p-value*
	n = 2,117	n = 3,366	
LDL-C, mean ± SD	92.7 ± 31.8	100.6 ± 35.1	<0.001
<70	23.6	15.7	<0.001
70–99	40.7	39.8	
100–129	24.5	28.7	
130 or higher	11.2	15.8	
Non-HDL-C, mean ± SD	121.6 ± 37.5	126.9 ± 37.2	<0.001
<100	29.8	23.4	<0.001
100–129	34.2	35.5	
130–159	22.3	24.4	
160 or higher	13.7	16.7	
TG, mean ± SD	152.2 ± 109.7	135.1 ± 83.4	<0.001
<150	62.1	70.0	<0.001
150–199	17.7	15.4	
200–249	9.8	6.7	
250 or higher	10.4	7.9	
HDL-C, mean ± SD	42.7 ± 12.2	46.4 ± 13.6	<0.001
<40 for male and <50 for female	53.3	39.6	<0.001

^*^Proportions were calculated based on the number of patients without missing value of that lipid parameter.

### Residual Risk Factors for Major Adverse Cardiovascular Events

The enrolled patients had a median follow-up of 2.7 years. Overall, the incidence rates of recurrent MACE per 100 person-years were 1.6 (95% CI 1.2–1.9) in the diabetic group and 1.0 (0.8–1.2) in the nondiabetic group (*p* = 0.68). Furthermore, the incidence rate per 100 person-years were 1.5 (1.1–1.9) and 1.7 (1.1–2.4) in men and women with diabetes, respectively, compared with 1.0 (0.8–1.3) in nondiabetic men and 1.0 (0.7–1.5) in nondiabetic women.

The adjusted risk of cardiovascular events was estimated using Cox proportional hazards modeling. In the diabetic ASCVD population, the risk of recurrent MACE was significantly increased with NYHA class I-II heart failure (hazard ratio [HR] 2.23 [95% CI 1.31–3.77]), and CKD stage 4–5 (vs. stage 1–2: HR 3.03 [1.49–6.16]), and decreased with use of beta blockers (HR: 0.63 [0.40–0.99]) (Table 3). Increased non-HDL-C levels were associated with a stronger event risk (HR 2.48 [1.10–5.59], 4.29 [1.91–9.65] and 4.59 [1.99–10.57] for non-HDL-C 100–129, 130–160 and ≥160 mg/dL respectively, vs. non-HDL-C < 100 mg/dL), as compared to using LDL-C as a lipoprotein level indicator in the model (top part of Table 4). (The Kaplan-Meier curves of MACE by different levels of non-HDL-C in diabetic and nondiabetic ASCVD patients were provided as online Supplementary Information.) There was a trend that higher LDL-C and TG level were associated with increased risk of events; however, only LDL-C level ≥130 mg/dL and TG level ≥250 mg/dL significantly increased the risk of major cardiovascular events (HR 2.55 [1.17–5.53] and 2.10 [1.13–3.92], respectively). Among nondiabetic ASCVD individuals, history of MI (HR 2.44 [1.27–4.69]), NYHA class I-II heart failure (HR 1.97 [1.17–3.31]), and CKD stage 4–5 (HR 3.02 [1.18–7.69]) were the significant independent predictor of MACE. None of the lipid panel parameters was significantly associated with recurrent cardiovascular events in patients without DM.

**Table 3:** Multivariable Cox proportional hazards model for predicting MACE in diabetic and nondiabetic ASCVD patients (Model for LDL-C).

Parameter	DM patients (n = 2,117)		Non-DM patients (n = 3,366)	
	Hazard ratio (95% CI**)**	*p*-value	Hazard ratio (95% CI)	*p-*value
Age	1.02 (1.00–1.04)	0.12	1.02 (1.00–1.04)	0.07
Male (vs. female)	0.88 (0.49–1.56)	0.66	0.75 (0.44–1.27)	0.28
BMI (kg/m^2^) (vs. 23 −<27.5)				
<23	1.31 (0.74–2.32)	0.35	1.53 (0.95–2.46)	0.08
≥27.5	0.99 (0.60–1.65)	0.97	0.93 (0.56–1.56)	0.79
Systolic blood pressure (mmHg)	1.01 (1.00–1.02)	0.11	1.00 (0.99–1.01)	0.66
HbA1c (%)	1.04 (0.90–1.19)	0.61	—	—
Cigarette smoking (vs. no smoking)	1.52 (0.88–2.60)	0.13	1.37 (0.86–2.17)	0.18
History of myocardial infarction	0.98 (0.54–1.79)	0.95	2.44 (1.27–4.69)	<0.01
Previous coronary or LEAD intervention	1.11 (0.67–1.85)	0.68	1.55 (0.99–2.44)	0.06
History of ischemic stroke or TIA	1.03 (0.58–1.84)	0.92	1.76 (1.00–3.11)	0.05
Hypertension	1.27 (0.70–2.30)	0.43	0.94 (0.59–1.49)	0.78
Heart failure (NYHA class I-II)	2.23 (1.31–3.77)	<0.01	1.97 (1.17–3.31)	<0.05
Chronic kidney disease (vs. Stage 1–2)				
Stage 3	1.44 (0.85–2.47)	0.18	1.37 (0.85–2.20)	0.20
Stage 4–5	3.03 (1.49–6.16)	<0.01	3.02 (1.18–7.69)	<0.05
With antiplatelet therapy	0.88 (0.47–1.66)	0.69	0.94 (0.54–1.65)	0.83
With ARBs or ACE inhibitors	0.68 (0.44–1.06)	0.09	1.33 (0.87–2.03)	0.19
With beta blockers	0.63 (0.40–0.99)	<0.05	0.70 (0.46–1.06)	0.09
With OHAs	1.42 (0.88–2.30)	0.15	—	—
With insulin	1.69 (0.74–3.85)	0.21	—	—
Statin intensity (vs. moderate-intensity)				
No statin use	0.92 (0.55–1.56)	0.76	1.35 (0.85–2.13)	0.20
Low-intensity	0.91 (0.43–1.89)	0.79	0.83 (0.39–1.76)	0.62
High-intensity	0.53 (0.13–2.21)	0.38	−*	—

*The hazard ratio could not be estimated because no event occurred in the 107 non-DM patients taking high-intensity statin therapy. ^†^These multivariable models included LDL-C levels and all the variables listed in this table. The adjusted hazard ratios for the association between LDL-C levels and recurrent MACE are listed in Table 4. ACE = angiotensin-converting enzyme; ARB = angiotensin II receptor blocker; LEAD = lower extremity arterial disease; NYHA = New York Heart Association; OHA = oral hypoglycemic agent; TIA = transient ischemic attack.

**Table 4:** Comparing the ability of various lipid parameters to predict MACE in diabetic and nondiabetic ASCVD patients.

Among all included patients						
Lipid panel parameter in the model^†^	DM patients (n = 2,117)			Non-DM patients (n = 3,366)		
	Adjusted hazard ratio	95% CI	*p*-value	Adjusted hazard ratio	95% CI	*p*-value
LDL-C level (vs. <70 mg/dL)						
70–99	1.49	0.75–2.96	0.26	0.78	0.45–1.34	0.36
100–129	1.99	0.97–4.07	0.06	0.82	0.46–1.47	0.51
130 or higher	2.55	1.17–5.53	<0.05	0.59	0.28–1.26	0.17
Non-HDL-C level (vs. <100 mg/dL)						
100–129	2.48	1.10–5.59	<0.05	1.03	0.62–1.73	0.90
130–159	4.29	1.91–9.65	<0.001	0.91	0.51–1.62	0.74
160 or higher	4.59	1.99–10.57	<0.001	0.73	0.35–1.49	0.38
TG level (vs. <150 mg/dL)						
150–199	1.20	0.66–2.20	0.55	0.93	0.54–1.61	0.80
200–249	1.69	0.85–3.35	0.13	0.52	0.16–1.71	0.28
250 or higher	2.10	1.13–3.92	<0.05	0.50	0.18–1.37	0.18
Higher HDL-C level						
(vs. Lower HDL-C)*	0.85	0.52–1.40	0.52	1.07	0.69–1.64	0.76
**Among statin-treated patients**						
**Lipid panel parameter in the model** ^**†**^	**DM patients (n = 1,472)**			**Non-DM patients (n = 2,203)**		
	**Adjusted hazard ratio**	**95% CI**	***p*** **-value**	**Adjusted hazard ratio**	**95% CI**	***p*** **-value**
LDL-C level (vs. <70 mg/dL)						
70–99	1.72	0.76–3.87	0.19	0.77	0.39–1.55	0.47
100–129	1.88	0.77–4.58	0.17	0.87	0.40–1.89	0.73
130 or higher	2.63	1.06–6.56	<0.05	0.67	0.25–1.77	0.42
Non-HDL-C level (vs. <100 mg/dL)						
100–129	2.98	1.15–7.71	<0.05	1.14	0.58–2.22	0.71
130–159	4.33	1.59–11.78	<0.01	1.07	0.48–2.36	0.87
160 or higher	4.14	1.50–11.42	<0.01	0.92	0.37–2.30	0.85
TG level (vs. <150 mg/dL)						
150–199	1.28	0.55–2.99	0.57	1.03	0.50–2.15	0.93
200–249	2.37	0.87–6.47	0.09	1.07	0.28–4.12	0.92
250 or higher	3.39	1.29–8.90	<0.05	0.66	0.14–3.07	0.59
Higher HDL-C level						
(vs. Lower HDL-C)*	0.85	0.47–1.52	0.58	0.80	0.46–1.39	0.43

*Lower HDL-C was defined as <40 mg/dL for male and <50 mg/dL for female. ^†^Each model was adjusted for age, gender, BMI categories, systolic blood pressure, HbA1c (only in diabetic patient group), cigarette smoking history, history of myocardial infarction, ischemic stroke or transient ischemic attack, hypertension, heart failure, chronic kidney disease, previous coronary or lower extremity arterial disease (LEAD) intervention, use of statin therapy, antiplatelet therapy, angiotensin receptor blocker or angiotensin-converting enzyme inhibitor, beta blocker, oral hypoglycemic agents (OHAs) and insulin at study enrollment.

Furthermore, when we limited the subjects to those on statin treatment, the regression analyses yielded similar results (bottom part of Table 4). On-treatment non-HDL-C level remained significant and may be a stronger predictor of recurrent cardiovascular events in patients with DM; LDL-C level of ≥130 mg/dL and TG level of ≥250 mg/dL were also associated with a higher risk, but no significant relationship was found between HDL-C levels and recurrent MACE.

## Discussion

This study examined the residual risk related to recurrent MACE in ASCVD patients with a high prevalence under lipid-lowering therapy, particularly in the subpopulations of diabetic and nondiabetic individuals, so as to elucidate potential approaches to reduce the residual CVD risk in these subpopulations. The results indicated that lipid profiles (especially non-HDL-C), heart failure (specifically for NYHA class I-II since more severe disease were excluded from enrollment), advanced renal disease and nonusers of beta blockers are independent predictors of adverse cardiovascular outcomes in atherosclerotic patients with diabetes, whereas heart failure, advanced renal disease, and history of MI confer a higher risk for MACE in nondiabetic atherosclerotic patients.

Non-HDL-C level, but not LDL-C, was found to be the most significant predictor among the lipid profile for MACE in the patients with DM. It has been well known that patients with DM are approximately two to four times more likely to develop CVD than people without DM^8, 9^. Individuals with diabetes remain at a substantial residual risk for CVD despite lifestyle modifications and statin therapy^10^. In type 2 DM, insulin resistance results in an excessive efflux of free fatty acids from adipose tissue, and the glycation of circulating lipoproteins with hyperglycemia increases the atherogenic potential^11–13^. Diabetic dyslipidemia is most often characterized by a high serum TG concentration, low HDL-C concentration, and increased concentration of small dense LDL-C particles^14, 15^. LDL-C is a well-established causal factor for ASCVD^1, 2^; however, the role of TG lowering and HDL-C level enhancement in CVD prevention is more uncertain given limited data support^9^. Given that, for more than a decade, LDL-C has been viewed as the primary target of therapy by most of the lipid management guidelines^1, 2, 16^. Nonetheless, LDL-C does not account for the entire cohort of circulating atherogenic lipid particles. As non-HDL-C represents the full components of atherogenic lipids (LDL-C, very-low-density lipoprotein [VLDL], intermediate-density lipoprotein [IDL], and lipoprotein[a]) and is a surrogate marker for apolipoprotein B (Apo B) for routine clinical practice^17^, it has recently been gaining importance over LDL-C as cardiovascular risk marker in patients with ASCVD. Several studies have suggested that non-HDL-C is superior for risk prediction and might be a more effective target for lipid-lowering therapy, particularly in high-risk patients^18–21^. A meta-analysis of 233,455 patients showed that non-HDL-C is a more potent marker of CVD risk than LDL-C^22^. Therefore, the US National Lipid Association (NLA) Expert Panel has arrived at the consensus that non-HDL-C is superior to LDL-C because non-HDL-C is more predictive of ASCVD risk in observational studies. When non-HDL-C and LDL-C are discordant, the risk is more closely associated with non-HDL-C^23^. Our study was able to fill the literature gap that serum non-HDL-C level served as a more critical predicting factor for recurrent MACE, than LDL-C and TG level, in diabetic ASCVD patients under secondary prevention.

The current study also supports the recommendations that ASCVD patients with concomitant DM need stricter control of their lipid levels^14^. Our results indicated that lipid profiles significantly predicted the recurrent MACE in diabetic patients after controlling for the use of statin and its intensity. In contrast, these associations were not found in those without diabetes. In fact, the noticeable impact of lipid abnormalities in diabetic patients (vs. nondiabetic patients) has recently been demonstrated in the IMProved Reduction of Outcomes: Vytorin Efficacy International Trial (IMPROVE-IT) study^24^. The prespecified subgroup analysis showed that the addition of ezetimibe to simvastatin therapy, which resulted in a greater decrease of LDL-C level, could significantly reduce the cardiovascular events only in the diabetic patients. Based on this evidence, the 2016 Expert Consensus Decision Pathway recently released by the American College of Cardiology (ACC) have recommended to consider non-statin medications added to statin therapy in diabetic ASCVD patients whose LDL-C reduction is less than 50% on maximally tolerated statin, in order to achieve the treatment goal of LDL-C <70 mg/dL or non-HDL-C < 100 mg/dL^25^. Taken together, it is implicated that diabetic patients need more aggressive lipid control, and it requires more attention to treat diabetic dyslipidemia given the uniquely altered pathophysiology and lipid profile.

A few studies have assessed the risk factors for recurrent CVD in type 2 diabetic patients^26, 27^. These studies showed that age, male gender, and use of insulin, having a major ischemic heart disease event as the first event, and even a series of baseline patient profile parameters before the first event were independent predictors for recurrent CVD. In these studies, however, only a small proportion (1.8–25.5%) of the studied populations, with DM and established ASCVD, were receiving lipid-lowering treatment at baseline. In contrast, over 70% of our studied ASCVD populations were under statin therapy. To help with the interpretation, we also conducted a subgroup analysis only in the patients receiving statin therapy and found similar results. As lipid-lowering treatment continues to be strongly recommended for secondary prevention of established CVD, our study results are more pertinent and applicable to the nowadays’ patient cohorts with a higher prevalence of lipid-lowering treatment in accordance with the current standard of care.

In addition to lipid profile, our results highlighted advanced renal disease as an important risk factor for MACE particularly in diabetic patients, although there was no statistically significant interaction between DM and CKD (data not shown). It is in part consistent with prior studies which indicated that chronic renal failure amplifies the atherogenic risk, probably by changing lipoprotein profile^28^. A number of randomized controlled trials and observational studies have also demonstrated that the presence of renal disease increases the risk of cardiovascular disease and mortality in diabetic subjects^29, 30^. On the other hand, it is equally important to identify the risk factors of MACE in nondiabetic ASCVD patients. This study demonstrated that heart failure and advanced renal disease were the shared risk factors for MACE in atherosclerotic patients with and without DM; however, history of MI was a risk factor for MACE in nondiabetic ASCVD patients, while a protective effect of beta blockers was observed in diabetic patients. Patients with the risk factors require more aggressive treatment to further reduce their risks for adverse cardiovascular events or mortality.

This prospective observational study demonstrated the current lipid-lowering treatment status in patients with established CVD, prespecified subgroup by the presence or absence of DM, and offered the real-world data about how to improve patient management to further reduce MACE in secondary prevention. Moreover, most of the treatment guidelines for secondary CVD prevention were drawn from large-scale clinical trials primarily conducted in Caucasian populations, with limited evidence available for the Asian populations. Considering the possibility of ethnical difference, one strength of this prospective study is that it was conducted with a large-scale registry of the patients with atherosclerotic diseases in many regions in Taiwan, hence enhancing the sample representativeness of ethnic Chinese population and minimizing the concerns of restricted sample size.

However, the study has several limitations. First, the patients enrolled in this study were treated mainly by the specialists in medical centers. Non-probability sampling was applied and it was not possible to ensure that the enrollment of patients was consecutive. In addition, it is noteworthy to mention that in the present study subjects had a lower incidence rate of recurrent MACE probably owing to the high percentage of lipid-lowering therapy^26, 27^. Our study cohort could represent a less vulnerable patient group also because patients were excluded from the T-SPARCLE Registry or analysis if they had dialysis or advanced heart failure (NYHA class III-IV), or if they recently experienced a stroke, acute coronary syndrome, or coronary revascularization procedure. Therefore, the study results may not reflect the patient management practices for treating atherosclerotic diseases across Taiwan, and generalization of the results beyond the study setting warrants caution. Second, we have no accurate or detailed information about patient’s complete medical history, duration of secondary prevention therapies, medication adherence, lifestyle modifications, and laboratory data. Moreover, the T-SPARCLE Registry was implemented since 2009 and we would need a longer patient follow-up to estimate the 10-year cumulative incidence of cardiovascular events. Also, we are not able to evaluate the event predictive ability of Apo B, which is not readily available for routine clinical use but has been identified as a superior marker over non-HDL-C in many studies including the INTERHEART study conducted in multiple countries^31^.

In conclusion, in atherosclerotic patients having a high prevalence of lipid-lowering therapy, heart failure, advanced renal disease, without beta blocker use, non-HDL-C, LDL-C, and TG are residual risk factors for adverse cardiovascular outcomes in those with diabetes, whereas heart failure, advanced renal disease, and history of MI confer a higher risk in nondiabetics. The study results implicated that ASCVD patients with concomitant diabetes need stricter control of their lipid profile, particularly non-HDL-C levels, to minimize the residual risk for cardiovascular morbidity and mortality.

## Methods

### Data Source (the T-SPARCLE Registry)

The study was conducted using the follow-up data as of 2015 March from a multi-center prospective observational registry study, the Taiwanese Secondary Prevention for patients with AtheRosCLErotic disease (T-SPARCLE) Registry, which involves 16 medical centers across all regions in Taiwan^32^. Briefly, the registry study attempts to recruit and follow up a large population of patients with ASCVD who have been receiving secondary prevention therapy, with a special focus on lipid management, so as to assess the treatment patterns, goal attainment, factors influencing treatment, and the associated clinical outcomes. Patients were enrolled if he or she fulfilled all the following criteria: (1) age >18 years; (2) with stable ASCVD (i.e., coronary atherosclerosis, cerebrovascular disease, or peripheral atherosclerosis—the detailed definitions can be found in previous publication^32^); (3) willing to follow National Cholesterol Education Program (NCEP) Therapeutic Lifestyle Change or similar cholesterol-lowering diet; (4) if female and receiving hormone therapy, a stable regimen was maintained from at least 8 weeks prior to enrollment and continued throughout the study; and (5) signed the inform consent. Individuals were excluded if having hemodynamically significant valvular or congenital heart diseases, heart failure in the New York Heart Association (NYHA) functional class III-IV, life-threatening malignancy, neurocognitive or psychiatric condition which prevents reliable clinical data collection, undergoing immunosuppressive therapy, or any condition or situation which, in the opinion of the investigator, might be not suitable for this registration. Patients were not allowed to participate if they had experienced an acute stroke, acute coronary syndrome, or coronary revascularization procedure within the past 3 months, or had been scheduled to undergo coronary bypass graft surgery or valvular surgery before study enrollment. Eligible patients who fulfilled the enrollment criteria at the screening visit would be followed every year for a total of 5 years, and every 2 years thereafter. The study protocol conforms to the ethical guidelines of the 1975 Declaration of Helsinki as reflected in a priori approval by the Joint Institutional Review Board, Taiwan, for recruiting patients in each participating hospital (JIRB number 09-S-015).

### Patient Selection and Data Collection

Patient baseline characteristics were collected at the registry enrollment. Clinical endpoints, vital signs, use of lipid-lowering agents, concurrent medications, adverse events, and laboratory data were recorded at enrollment and each follow-up. The data were obtained mainly from medical records but also through patient self-report if formal medical record was not available. Particularly, diagnosis of diabetes, medication use at enrollment, and recurrent CVD events were registered based on medical records, and the diagnoses were made and ascertained by patients’ primary care physicians. Non-high-density lipoprotein cholesterol (non-HDL-C) levels were calculated by subtracting the HDL-C from the total cholesterol levels. In this study, patients were included if they were recruited between January 2010 and August 2014. For the purpose of this analysis, we further excluded patients if: (1) no outcome follow-up visit after enrollment; (2) atherosclerotic disease type or history of diabetes was unknown; (3) at least two statins were taken at study enrollment; or (4) they had end-stage renal disease receiving maintenance dialysis.

### Study Outcome Endpoint

The primary endpoint of this study was the time to the first MACE since enrollment. MACE is a composite endpoint which includes cardiovascular death, nonfatal stroke (ischemic stroke, hemorrhagic stroke, transient ischemic attack [TIA], or vertebrobasilar insufficiency [VBI]), nonfatal myocardial infarction (MI, including non-ST-elevation MI or ST-elevation MI), or cardiac arrest with resuscitation.

### Statistical Analysis

Patients were stratified into two groups: with and without a known diagnosis of DM at the enrollment. Categorical variables are presented as percentage and continuous variables as mean ± standard deviation (SD). Chi-squared test was used to compare proportions and student’s t- test was applied to compare the difference in the mean values of continuous variables between two groups. Survival analysis was conducted separately in patients with and without DM, and multivariable Cox proportional hazards regression modeling was used to identify independent predictors of MACE occurrence while adjusting for covariates; hazard ratio (HR) and 95% confidence interval were estimated. The potential clinical variables such as age, gender, body mass index (BMI), systolic blood pressure (SBP), HbA1c, smoking status, medical history (hypertension, heart failure, MI, ischemic stroke or TIA, and CKD), previous interventions performed for coronary or peripheral artery, statin use and intensity at enrollment, other medication use (antiplatelet therapy, angiotensin-converting enzyme [ACE] inhibitors or angiotensin II receptor blockers [ARBs], beta blockers, oral hypoglycemic agents [OHAs], insulin) and lipid profiles were included in the analyses. The covariates of HbA1c and use of OHAs and insulin were considered in the regression model only for the diabetic group. The categorization of BMI (<23, 23− <27.5, and ≥27.5 kg/m^2^) was based on the WHO’s recommended cut-off points for determining public health and clinical action in the Asian populations^33^. Glomerular filtration rate (GFR) was calculated^34^ and the presence of CKD was further classified as GFR between 30–60 ml/min, and 30 ml/min or less. The intensity of statin therapy (low, moderate, and high) was defined based on the average extent of low-density lipoprotein cholesterol (LDL-C) lowering effect (by <30%, 30 to <50%, and ≥50%, respectively) with its daily dose^2^. Data analyses were performed using the Statistical Analysis System (SAS) version 9.4 (SAS Institute, Cary, NC), and a two-sided *p*-value of <0.05 was considered statistically significant.

### Missing Data Imputation

At registry enrollment, about 5% of the enrolled patients had missing data on BMI, SBP and triglyceride (TG), and 8–12% in LDL-C, HDL-C, non-HDL-C, or GFR. HbA1c data were missing in 22% of the diabetic participants. To handle the missing data, we used multiple imputation (by PROC MI procedure in SAS), which is considered the most effective way of treating missing data and may provide unbiased results with the least influence by the proportion and mechanism of missingness^35^. Multiple imputation acknowledges the uncertainty associated with the imputed values by generating a set of *m* plausible values (in this study, *m* = 20) for each unobserved data point, using alternative configurations of covariates. The imputation step resulted in twenty complete data sets, each with one unique estimate of the missing values. After imputation, we fit Cox proportional hazards model for each complete data set, and then used PROC MIANALYZE procedure in SAS to combine results from the twenty Cox models (the pooled standard error of the parameter estimate incorporates the uncertainty due to the missing data treatment).

## Electronic supplementary material

Supplementary material
