# Supplementary material for: Residual Risk Factors to Predict Major Adverse Cardiovascular Events in Atherosclerotic Cardiovascular Disease Patients with and without Diabetes Mellitus
Source: Sci Rep. 2017 Aug 23;7:9179. doi: 10.1038/s41598-017-08741-0 (PMC5569020; doi:10.1038/s41598-017-08741-0)

Supplementary Information

Article title: Residual Risk Factors to Predict Major Adverse Cardiovascular Events in Atherosclerotic Cardiovascular Disease Patients with and without Diabetes Mellitus

Journal: ***Scientific Reports***

Authors: Fang-Ju Lin, Wei-Kung Tseng, Wei-Hsian Yin, Hung-I Yeh, Jaw-Wen Chen, Chau-Chung Wu

Affiliation and e-mail address of corresponding author:

Chau-Chung Wu, MD, PhD

Attending Physician, Department of Internal Medicine (Cardiology Section), National Taiwan University Hospital, Taipei, Taiwan

Professor, Graduate Institute of Medical Education & Bioethics, College of Medicine, National Taiwan University, Taipei, Taiwan

Email: chauchungwu@ntu.edu.tw

Supplementary Figure S1 Flow diagram of patient enrollment

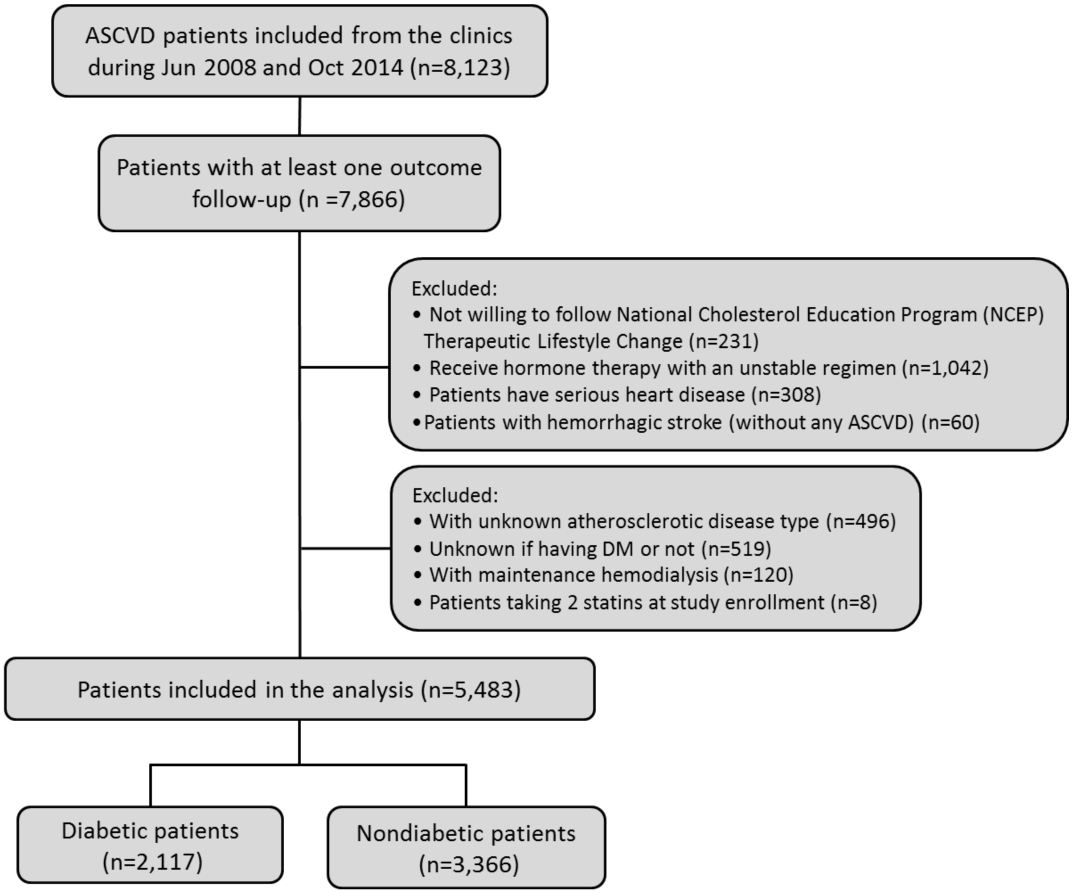

Note: Patients may be excluded by more than one reason in each of the exclusion box.

Supplementary Figure S2-1 Kaplan-Meier curve of major adverse cardiovascular event (MACE) by non-HDL-C levels in diabetic ASCVD patients

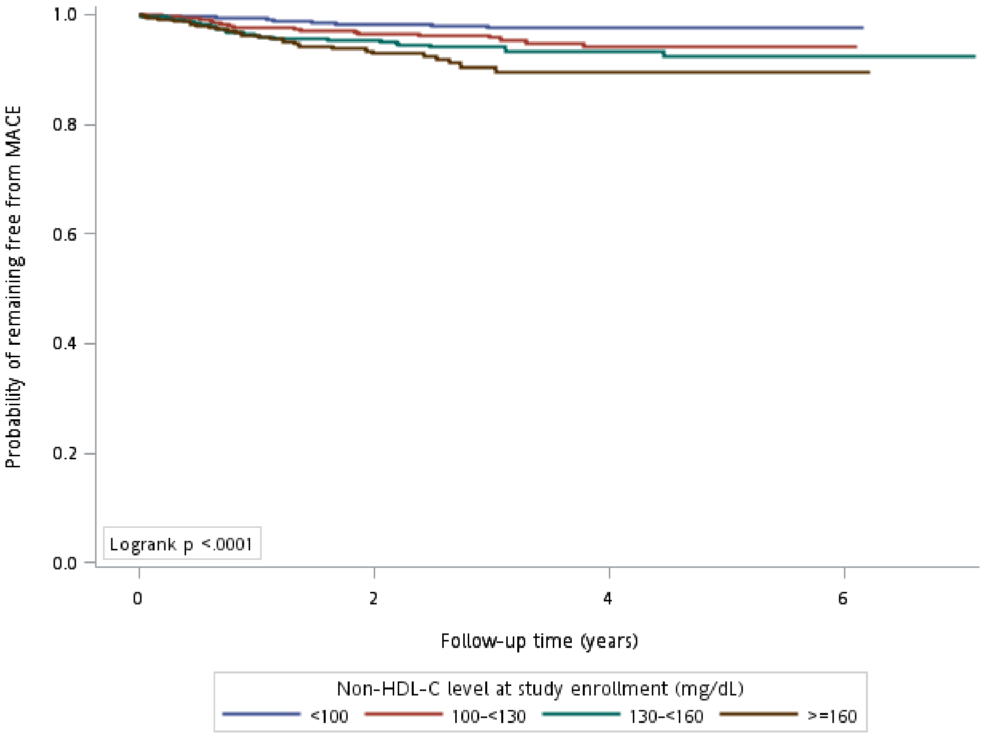

Supplementary Figure S2-2 Kaplan-Meier curve of major adverse cardiovascular event (MACE) by non-HDL-C levels in nondiabetic ASCVD patients

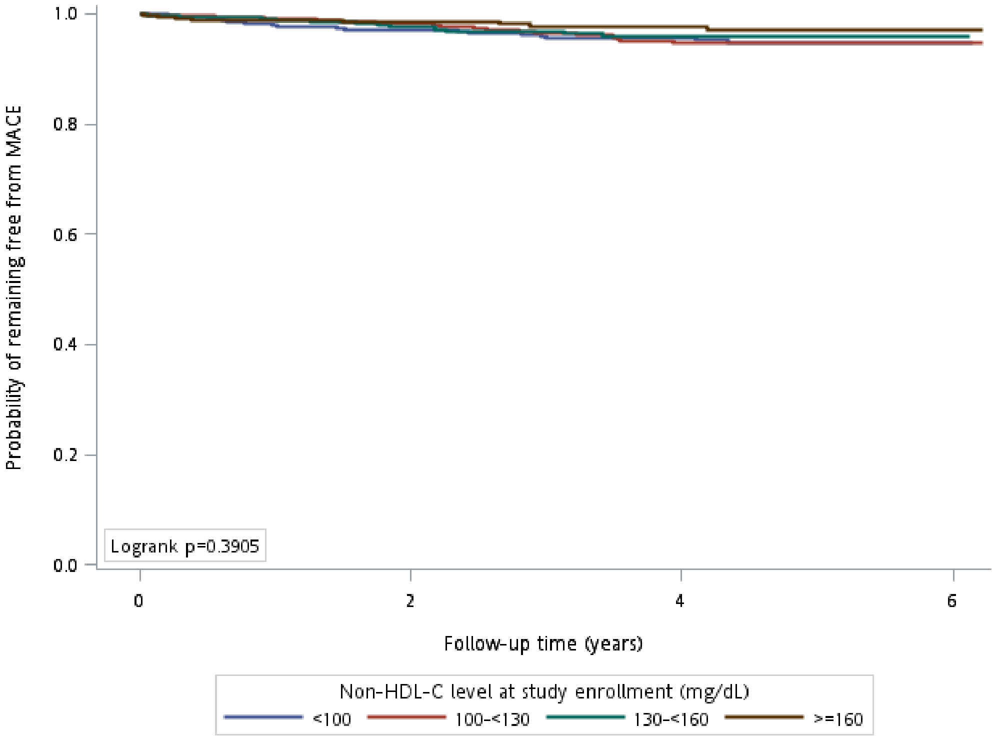

Supplement: Supplementary file 1 — Supplementary material [file 41598_2017_8741_MOESM1_ESM.pdf]
